# Clinical and psychological status of patients with different types of juvenile arthritis

**DOI:** 10.1186/1546-0096-12-S1-P102

**Published:** 2014-09-17

**Authors:** Natalia Stepanenko, Irina Nikishina, Tatyana Shelepina

**Affiliations:** 1 Psychology, «Research Institute of Rheumatology named after V.A. Nasonova», Moscow, Russian Federation; 2 Federal State Budgetary Institution «Research Institute of Rheumatology named after V.A. Nasonova», Moscow, Russian Federation

## Introduction

Juvenile arthritis (JIA) is chronic potentially disabling disease, which may form certain psychological features in children

## Objectives

The purpose is to reveal difference in psychological status of children depending on the clinical type of JIA.

## Methods

clinical interview; Lüscher 8-color test; Spielberger-Khanin test; CMAS (A. Prikhozhan adaptation); family drawing test; non-existent animal test, house tree man test; pathodiagnostic test

## Results

A tendency for higher level of personal anxiety is found in patients with oJA (33.3%), to a lesser extent in patients with pJA (20.7%) and sJA (17.6%). The patients have low social adaptation (16.7%; 11.3%; 5.9% correspondingly) and signs of school stress (11.1% with oJA; 5.7% with pJA; 0% with sJA). Cognitive disorders (23.5% with sJA; 11.1% with oJA; 7.5% with pJA) and aggression (11.8% with sJA; 7.5% with pJA; 0% with oJA) are more frequent in patients with JA. Communicative disorders are equally usual in patients with sJA (35.3%) and pJA (32%) and found in 22.2% of patients with oJA. Dissatisfaction with appearance is more frequent in patients with sJA (11.8%) and oJA (11.1%) than in patients with pJA (7.5%). Chronic low mood slightly prevails in patients with pJA (13.2%) over sJA (11,8%) and occurs in 5.5% of patients with oJA. Different neurotic fears are found with virtually the same frequency in patients with pJA (28.3%) and oJA (27.8%) and also in 5.9% of patients with sJA. Domestic stress is equally felt by patients with all JA types (44.4% with oJA; 41.5% with pJA; 35.3% with sJA). Hospital adaptation is harder for patients with oJA (5.5%) and pJA (3.8%) than for patients with sJA (0%).

## Conclusion

Children with different types of JIA have been found to have certain differences in psychological status. Chronic low mood is more frequent in patients with pJA than in case of other types of the disease. Cognitive disorders and aggression are more frequent in patients with sJA. Patients with oJA are characterized by more frequent symptoms of personal anxiety, low social adaptation and school stress. Domestic stress indicators can be noted in patients with all types of JIA. Clinical psychological status of patients with JIA needs further study with larger material to reveal stable tendencies and develop psychocorrective programs.

## Disclosure of interest

None declared.

